# Lnc-HIBADH-4 Regulates Autophagy-Lysosome Pathway in Amyotrophic Lateral Sclerosis by Targeting Cathepsin D

**DOI:** 10.1007/s12035-023-03835-5

**Published:** 2023-12-22

**Authors:** Jingxuan Huang, Yujiao Yu, Dejiang Pang, Chunyu Li, Qianqian Wei, Yangfan Cheng, Yiyuan Cui, Ruwei Ou, Huifang Shang

**Affiliations:** Department of Neurology, Laboratory of Neurodegenerative Disorders, Rare Diseases Center, West China Hospital, Sichuan University, No.37, Guoxue Lane, Chengdu, 610041 Sichuan China

**Keywords:** ALS, Lnc-HIBADH-4, CTSD, miR-326

## Abstract

Amyotrophic lateral sclerosis (ALS) is the most prevalent and lethal class of severe motor neuron diseases (MND) with no efficacious treatment. The pathogenic mechanisms underlying ALS remain unclear. Nearly 90% of patients exhibit sporadic onset (sALS). Therefore, elucidating the pathophysiology of ALS is imperative. Long non-coding RNA (lncRNA) is a large class of non-coding RNAs that regulate transcription, translation, and post-translational processes. LncRNAs contribute to the pathogenesis of diverse neurodegenerative disorders and hold promise as targets for interference in the realm of neurodegeneration. However, the mechanisms of which lncRNAs are involved in ALS have not been thoroughly investigated. We identified and validated a downregulated lncRNA, lnc-HIBADH-4, in ALS which correlated with disease severity and overall survival. Lnc-HIBADH-4 acted as a “molecular sponge” regulating lysosomal function through the lnc-HIBADH-4/miR-326/CTSD pathway, thereby impacting autophagy-lysosome dynamics and the levels of cell proliferation and apoptosis. Therefore, this study discovered and revealed the role of lnc-HIBADH-4 in the pathogenesis of ALS. With further research, lnc-HIBADH-4 is expected to provide a new biomarker in the diagnosis and treatment of ALS.

**Supplementary Information:**

The online version contains supplementary material available at 10.1007/s12035-023-03835-5.

## Introduction

Amyotrophic lateral sclerosis (ALS) constitutes the most prevalent and lethal class of motor neuron disorders (MND) typified by heterogeneous presentations, generally rapid progression and high mortality [1, 2]. The mechanisms underlying motor neuron death remain widely unclear but implicate apoptosis, necroptosis, autophagy, and ferroptosis [3–5]. Most patients of ALS are sporadic, without specific pathologic gene variants in the process of disease. It is imperative to investigate the molecular mechanisms regulating ALS initiation and progression and identifying novel therapeutic targets improving the clinical outcome [6].

Recently, genome-wide association study (GWAS) discovered several non-coding variants in ALS patients [7]. Long non-coding RNAs (lncRNAs) constitute RNA molecules exceeding 200 nucleotides with limited protein-coding potential, regarded as the “dark matter” of biological functions in cells [8]. Recently, a large number of lncRNAs have come into focus using next-generation sequencing technology in the landscapes of neurological disorders, including neurodegenerative diseases [9]. Functions of lncRNAs range from epigenetic, transcriptional, to post-transcriptional levels [10]. It is reported that lncRNAs contribute to the initiation and progression of ALS [11], such as the interplays between lncRNAs and risk genes of ALS [12], or facilitates the misfolding protein formation [13]. It remains of interest to discover the unexplored role of lncRNAs in ALS. LncRNAs perform diverse functions in physiological and pathological processes, regarding as competing endogenous RNAs (ceRNAs) to eliminate inhibition of targeted gene mediated by miRNAs [10]. The mechanism whereby lncRNAs sponges to miRNAs has been extensively investigated and correlates with diverse disease pathophysiological progressions. Several miRNAs could serve as diagnostic and prognostic biomarkers for ALS [14–19]. Also, AAV-delivered miRNAs could provide potential for a targeted therapy for ALS patients carrying *SOD1* variant [20, 21]. Therefore, lncRNAs harbor considerable promise for mechanisms and therapies in ALS especially by functioning as molecular sponges for miRNAs.

The autophagy-lysosomal pathway participates in the pathogenesis of ALS [22]. As degradative compartments facilitating the elimination of cellular waste, lysosomes are critical for maintaining cellular homeostasis by the degradation of misfolded and toxic proteins [23]. Lysosomal dysfunction caused by variants of lysosomal-related genes is implicated in several neurodegenerative diseases, such as Alzheimer’s disease (AD), Parkinson’s disease (PD), as well as ALS [24–26]. Lysosomes need specific proteases to fulfill their function [27]. Cathepsin D (CTSD) is one of the proteases and highly expressed in the brain, which was confirmed critical for maintaining protein homeostasis in the brain, especially the homeostasis mediated by autophagy-lysosomal pathway [28, 29]. Previous studies reported that CTSD can serve as a target for miRNAs [30, 31]. However, the mechanisms of CTSD dysfunction and involvement in lysosome in ALS, especially whether lncRNA participates in miRNA/CTSD pathway remains unknown.

In order to figure out the role of lncRNAs in the progression of ALS, we expected to identify biomarkers of lncRNAs from peripheral blood mononuclear cells (PBMCs), due to their easy availability, and explore the biological function of lncRNAs in vitro. We discovered a novel lncRNA lnc-HIBADH-4 which was downregulated in PBMCs of ALS patients. Lnc-HIBADH-4 promoted proliferation while decreasing apoptosis in vitro. Moreover, lnc-HIBADH-4 acted as a miRNA sponge regulating the expression of CTSD, thereby inducing lysosomal activity, cell proliferation, and apoptosis. Therefore, our findings indicate that lnc-HIBADH-4 maintains cell homeostasis and provide a potential biomarker and therapy target for ALS.

## Material and Methods

### Clinical Samples

We obtained 60 PBMC samples from ALS patients, age- and sex-matched 40 health controls (HCs) and 40 PD patients from West China Hospital. Detailed clinical characteristics of patients in cohorts are provided in Supplementary Table 1. Research has been passed ethical approval from the Institutional Review Board of West China Hospital. All participants, patients and HCs, provided informed consent. The inclusion and exclusion criteria of participants were mentioned previously [11].

### PBMC Isolation from Venous Blood

A total of 5 ml venous blood was collected from patients and HCs. PBMCs were isolated from the Ficoll centrifugation (TBDscience, Tianjin, China) within 4 h after blood drawing. Phosphate buffer saline (PBS, Biosharp, China) was used to wash isolated PBMCs. The procedure of RNA extraction was described below.

### Cell Culture

Experiments in vitro were accomplished in human cervical cancer cell lines HeLa, besides, the human neuroblastoma cell lines SH-SY5Y (for RNA FISH and lnc-HIBADH-4 expression under stress stimulation) and human osteosarcoma cell lines U2OS (for cell infection of mRFP-GFP-LC3 vector) were also used in the research. These cell lines were obtained from the National Collection of Authenticated Cell Cultures (Shanghai, China). SH-SY5Y cells were cultured in DMEM/F12 medium (Thermo Fisher Scientific, Waltham, USA) with 10% fetal bovine serum (FBS, Thermo Fisher Scientific) and 1% penicillin/streptomycin solution (Thermo Fisher Scientific). HeLa and U2OS cells were maintained in DMEM with 10% FBS (ExCell Bio, Shanghai, China) and 1% penicillin/streptomycin solution. All cell lines were cultured at 37 ℃ incubators with 5% CO_2_.

### Reverse Transcription and Quantitative Real-Time PCR (RT-qPCR)

Total RNA was isolated using the TRIzol reagent (Thermo Fisher Scientific). Then, RNA was performed reverse transcription to cDNA by the Prime-Script™ RT Reagent Kit (Takara). Then, qPCR program was conducted on Applied Biosystems 7500 and 7500 Fast Real-Time PCR Systems (Thermo Fisher Scientific) with TB Green® Premix Ex Taq™ II (Takara). Cycling conditions were mentioned in previous studies [11]. The 2-^ΔΔCt^ method was performed for the analysis of RNA expression. GAPDH was used as internal reference for lncRNA, mRNA, and U6 (GeneCopoeia, USA) was used for miRNA in qPCR. Primer sequences are provided in Supplementary Table 2.

### RNA FluorescenceIn SituHybridization (RNA FISH)

HeLa and SH-SY5Y cells were cultured in 12-well plates with slides placed in the plates in advance at 37 ℃ overnight, and then performed cell fixation with 4% paraformaldehyde (PFA, Beyotime, Shanghai, China) for 15 min at room temperature. Subsequently, cells were cultured with 2–4 μM Streptavidin Cy3-labeled lnc-HIBADH-4 FISH probe (Gene Pharma, Shanghai, China), and Cy3-labeled negative/positive control probe at 37 ℃ for 12–16 h. After hybridization, 4,6-diamino-2-phenylindole (DAPI, Thermo Fisher Scientific) was used for nuclear staining away from light. Images were collected and taken at 100 × magnification with a fluorescence microscope (Nikon Ti2-U, Tokyo, Japan).

### Lentiviral Vectors and Cell Infection

U2OS cells were cultured into 6-well plates with 2 × 10^5^ cells per well. We infected cells with 20 μl mRFP-GFP-LC3 lentivirus (Genechem, Shanghai, China) and 40 μl infectious reagent in the next day. We replaced the medium at 24 h after cell infection and observed the infectious efficiency at 48 h and 72 h after cell infection. We added puromycin with 2.0 μg/ml and screened stable cell line after 48 h, then reduced puromycin to 0.5 μg/ml for screening in 2 weeks, and surviving cells were observed under the fluorescence microscope for further research.

### Cell Transfection

The short interfering RNA (siRNA) of lnc-HIBADH-4, miR-326 mimics, inhibitor and negative control were purchased from Gene Pharma (Shanghai, China). The sequences of siRNA were as follows: lnc-HIBADH-4 siRNA39: 5′-GCAUGGUGAUGAACCACAATT-3′; lnc-HIBADH-4 siRNA162: 5′-GGAGGAGGAAGAACUGAAATT-3′; siNC: 5′-UUCUCCGAACGUGUCACGUTT-3′. The pcDNA3.1 vector expressing lnc-HIBADH-4 and their empty vector were synthesized by Genechem (Shanghai, China). Cells (1 × 10^5^ per well for 12-well plate, 1 × 10^6^ per well for 6-well plate) were transfected with plasmids, siRNA or miRNA mimics/inhibitor using JetPRIME (Polyplus, Illkirch, France) followed by instructions with the concentration 0.8 μg (12-well plate) and 1.6 μg (6-well plate). The transfected cells were collected for further analysis after 48 to 72 h following preliminary experiment.

### Cell Proliferation Assays

Cell Counting Kit-8 (CCK-8) assay (Bimake, Shanghai, China) was used to detect cell viability. Cells were cultured into plates and treated according to experiment requirements. Then 10 × volume of CCK-8 reagent was supplemented into the cell culture medium for 2 h following preliminary experiment at 37 ℃. The microplate reader Synergy H1 (Bio-Tek, California, USA) was performed to read absorbance at 450 nm for cell viability analysis.

### Cell Apoptosis Assays

We used the Annexin V FITC-PI staining assay by flow cytometry to examine apoptosis. Cells (1 × 10^6^ per well) were pre-treated with H_2_O_2_ at 200 or 500 μM for 2, 4, 12 h, and staurosporine (STS) at 1 or 2 μM for 2, 4, and 12 h. After collecting cells, we used Annexin V-FITC/PI (silenced groups) or Annexin V-Alexa Fluor 647/PI (overexpressed groups) apoptosis detection Kit (YEASEN) at room temperature for 15 min away from light. Stained cells were detected by BD/FACS Aria III Flow cytometry (BD Biosciences, Piscataway, USA) within 1 h after dyeing.

### Western Blot

HeLa cells were collected for total protein cell lysis buffer (Beyotime) supplemented with protease inhibitor cocktail and phosphatase inhibitor cocktails (MedChemExpress, New Jersey, USA). BCA protein assay kit (BOSTER, Wuhan, China) was subsequently used to detect protein concentration, and then, protein was denaturated in loading buffer. Then, protein samples were loaded on a sodium dodecyl sulfate polyacrylamide gel electrophoresis (SDS-PAGE) for separation of protein with different molecular weight. Then the protein in SDS-PAGE was transferred to polyvinylidene fluoride (PVDF) membrane with a pore size of 0.22 μm, followed by 5% skim milk blocking. We then cut the PVDF membrane at the adequate size for protein of interest and incubated the strips into primary antibody at 4 ℃ overnight. The information of primary antibodies is provided in Supplementary Table 3. Then the strips were incubated with anti-rabbit secondary antibody or anti-mouse secondary antibody (Thermo Fisher Scientific; diluted ratio: 1:5000) for 1–2 h, and exposed to chemiluminescence (Millipore, Burlington, MA, USA and ECL, ThermoFisher Scientific) for image acquisition in ChemiDoc Touch Imaging System (BIO-RAD, USA).

### LysoTracker and LysoSensor Staining

LysoTracker Red DND-99 (YEASEN) is a red fluorescent-labeled lysosomal probe that can activate red fluorescence for acidic organelles. LysoSensor Yellow/Blue DND-160 (YEASEN) is a probe for the determination of lysosome depending on pH. LysoSensor probe produces blue fluorescence in a neutral environment, but blue fluorescence dims when the environment becomes more acidic. Therefore, lysosome acidic environment can be marked by LysoTracker Red DND-99 and LysoSensor Yellow/Blue DND-160 probe staining. Firstly, HeLa cells were prepared in the cover slide. LysoTracker Red DND-99 probe and LysoSensor Yellow/Blue DND-160 probe were added followed by instructions, incubate in CO_2_ incubator at 37 ℃ for 30 min–2 h. Then, cells were observed under a fluorescence microscope (Nikon) at 40 × magnification.

### Dual Luciferase Reporter Assay

According to the predicted binding sites of miRNA and lncRNA/mRNA (RNA hybrid website: https://bibiserv.cebitec.uni-bielefeld.de/rnahybrid/submission.html/), the plasmids of lnc-HIBADH-4-wt-LUC, lnc-HIBADH-4-mut-miR-326-LUC, CTSD-wt-LUC and CTSD-mut-miR-326-LUC were constructed from Gene Pharma (Shanghai, China). These vectors transfections were carried out above. Cells (1 × 10^5^ per well) were plated into 12-well plates, and transfected vectors with miR-326 mimics/NC mimics in the next day. After the process with cell lysis buffer, firefly luciferase reaction detection and renilla luciferase reaction detection were carried out one after the other (Vazyme, Nanjing, China). Data was collected by the multimode microplate reader (Bio Tek, USA).

### Transcriptome Sequencing

RNA/DNA was purified with three repeated groups of NC siRNA, lnc-HIBADH-4 siRNA, NC overexpression, and lnc-HIBADH-4 overexpression. The library construction and mRNA sequencing were performed by UID-mRNA-seq PE-150, with 6G sequencing volume. After sequencing, we constructed data pre-processing, transcriptome expression analysis and variable splicing analysis. Gene expression level was calculated by fragments per kilobase of transcript sequence per millions base pairs sequenced (FPKM). Differential gene expression of siRNA/OE groups compared with each NC groups was analyzed by the DESeq software. KEGG analysis (http://www.genome.jp/kegg/) was performed to explore the biological functions of differentially expressed genes between siRNA/OE groups and each NC groups.

### Statistical Analyses

Data from 3–5 independently repeated experiments were conducted for statistical analysis. Statistical software SPSS 26.0 was used for data analysis, and GraphPad Prism 9 software was carried on the results delivering. The continuous data variables were represented by mean ± standard error of mean (SEM). Unpaired *T*-test (data were distributed normally) or Mann–Whitney *U* test (data were not normally distributed) were used to compare two groups of continuous variables, and one-way analysis of variance (ANOVA) was used to compare multiple groups of data. Pearson correlation analysis was performed to calculate correlation coefficient. Kaplan–Meier analysis and log-rank test were used for survival analysis. Pearson correlation analysis and survival analysis were performed from the online website (http://www.bioinformatics.com.cn/). The difference was considered statistically significant at 0.05.

## Results

### LncRNA lnc-HIBADH-4 Is Downregulated in ALS Patients

**Fig. 1:**
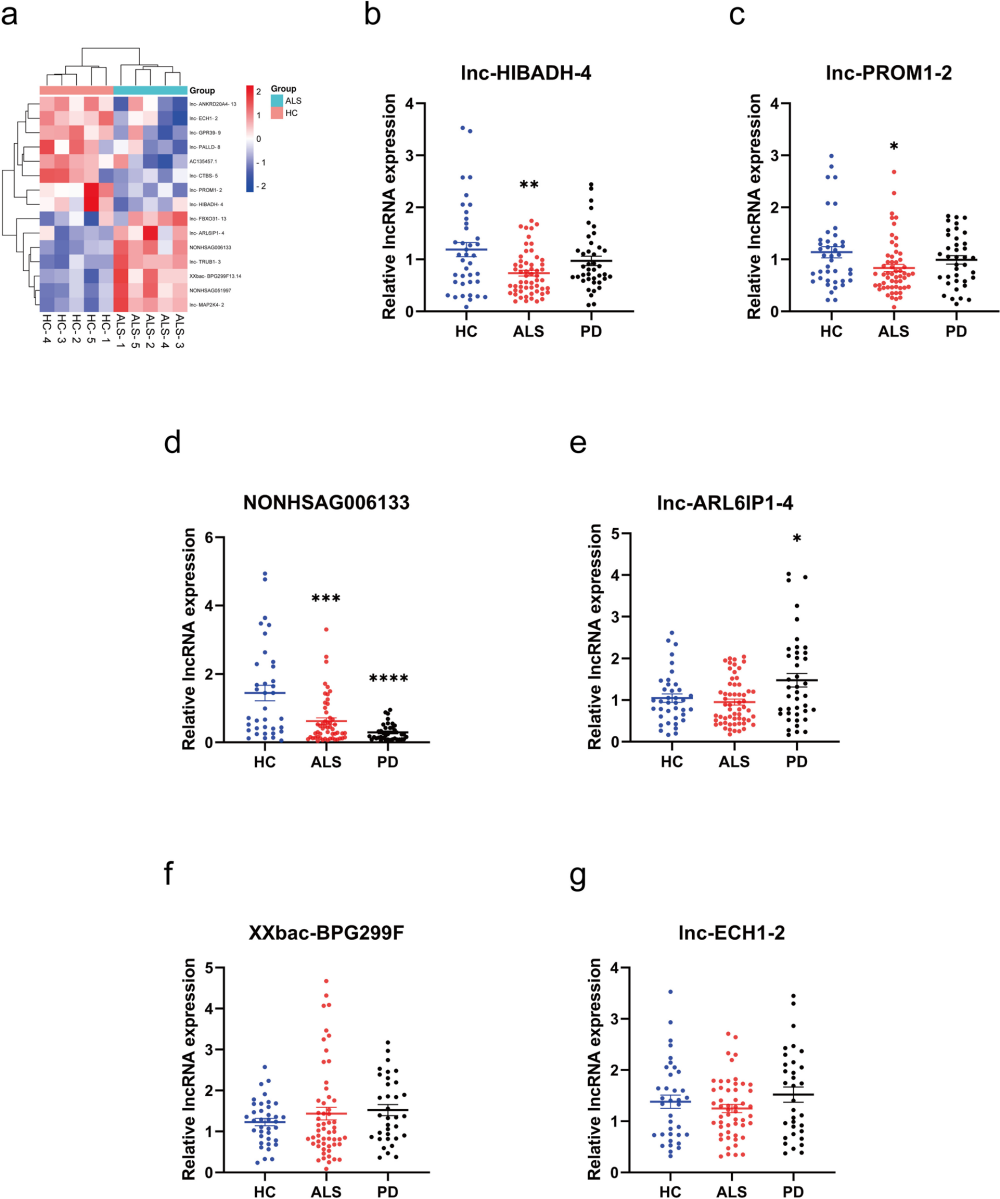
The validation of lncRNAs from Microarray by RT-qPCR. **a** Heatmap of Microarray from 5 sALS and 5 HC. **b** Lnc-HIBADH-4 expression in sALS (mean ± SEM, **P < 0.01 versus HC). **c** Lnc-PROM1-2 expression in sALS (mean ± SEM, *P < 0.05 versus HC). **d** NONHSAG006133 expression in sALS (mean ± SEM, ***P < 0.001 versus HC, ***P < 0.0001 between PD and HC). **e**–**g** Lnc-ALRIP1-4, XXbac-BPG299F, lnc-ECH1-2 expression in sALS (mean ± SEM)

To identify the role of lncRNAs in the process of ALS, we previously performed lncRNA microarray from 5 sporadic ALS patients and 5 age matched healthy control (HC) [11] (Fig. 1a). We then selected differentially expressed 6 lncRNAs in a validation cohort consisting of 60 sporadic ALS patients, 40 age-matched HCs, and 40 patients with PD as a neurological disorder control group. The information of lncRNAs was provided in Supplementary Table 4. Our analysis revealed that the expression of lnc-HIBADH-4 and lnc-PROM1-2 were specifically downregulated in sALS patients but showed no significant difference between PD patients and HCs, corresponding the results in Microarray (Fig. 1b, c). LncRNA NONHSAG006133 downregulated both in sALS and PD patients compared with HC (Fig. 1d), and other lncRNAs were found no significant difference between sALS and HCs (Fig. 1e–g).

**Fig. 2:**
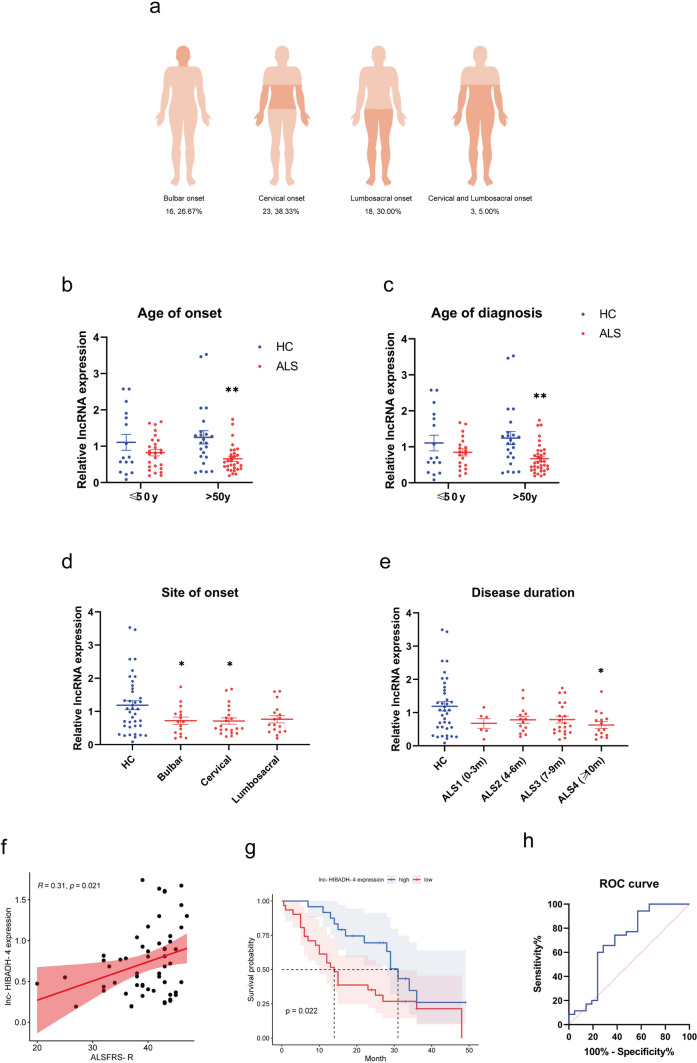
The correlation between lnc-HIBADH-4 and clinical characteristics of sALS. **a** Diagram in regard to site of onset in sALS patients of validation cohort. **b** Lnc-HIBADH-4 expression in sALS patients with different age of onset (mean ± SEM, **P < 0.01 in sALS with age of onset > 50 years versus HC > 50 years). **c** Lnc-HIBADH-4 expression in sALS patients with different age of diagnosis (mean ± SEM, **P < 0.01 in sALS with age of diagnosis > 50 years versus HC > 50 years). **d** Lnc-HIBADH-4 expression in sALS patients with different site of onset (mean ± SEM, *P < 0.05 in patients with bulbar or cervical onset versus HC). **e** Lnc-HIBADH-4 expression in sALS patients with disease duration (mean ± SEM, *P < 0.05 in patients with more than 10 months course of ALS versus HC). **f** Pearson correlation analysis of lnc-HIBADH-4 expression and ALSFRS-R score. **g** Survival analysis of sALS patients with relatively low and high expression of lnc-HIBADH-4 (Kaplan–Meier estimation and the log-rank test, the expression of lnc-HIBADH-4 lower than mean lnc-HIBADH-4 expression was defined as relatively low expression group, and vice versa). **h** ROC curve of lnc-HIBADH-4 in predicting ALS survival

Then, we correlated the expression of lncRNA and clinical characteristics of sALS patients. Among the 60 patients with sALS, they were divided into bulbar onset type (16, 26.67%) and spinal cord onset type (44, 73.33%) according to different initial locations. Among the spinal cord onset type, 23 cases (cervical onset, 38.33%) started from upper extremity, 19 cases (lumbosacral onset, 30.00%) started from lower extremity, and 3 cases (5.00%) started from both upper and lower extremity simultaneously (Fig. 2a). Notably, the downregulation of lnc-HIBADH-4 was more pronounced in patients over 50 years of age and those with onset of disease after the age of 50 (Fig. 2b, c). Furthermore, decreased expression of lnc-HIBADH-4 was associated with bulbar and cervical spinal cord onset of sALS, as well as longer disease duration (more than 10 months) (Fig. 2d, e). Moreover, we found a positive correlation between lnc-HIBADH-4 expression and ALSFRS-R score (Fig. 2f). Patients with lower expression of lnc-HIBADH-4 had significantly shorter survival times (Fig. 2g), and the area under the receiver operator characteristic (ROC) curve was 69.12% in the expression of lnc-HIBADH-4 to predict ALS survival (Fig. 2h). These findings demonstrated that lnc-HIBADH-4 was specifically downregulated in ALS patients and was associated with disease severity and survival. We also found the correlation between age of onset/diagnosis and the expression of lnc-PROM1-2 (Supplementary Fig. 1a, b). Lnc-PROM1-2 significantly downregulated in sALS patients with more than 10 months disease duration (Supplementary Fig. 1c). However, we failed to find the association between disease onset, severity, survival, and the expression of lnc-PROM1-2 (Supplementary Fig. 1d-f). According to these results, lnc-HIBADH-4 was found correlated with disease characteristics, especially disease severity and survival, so that we selected lnc-HIBADH-4 for subsequent functional experiments in vitro.

### Knockdown lnc-HIBADH-4 Inhibits Cell Proliferation and Promotes Apoptosis

**Fig. 3:**
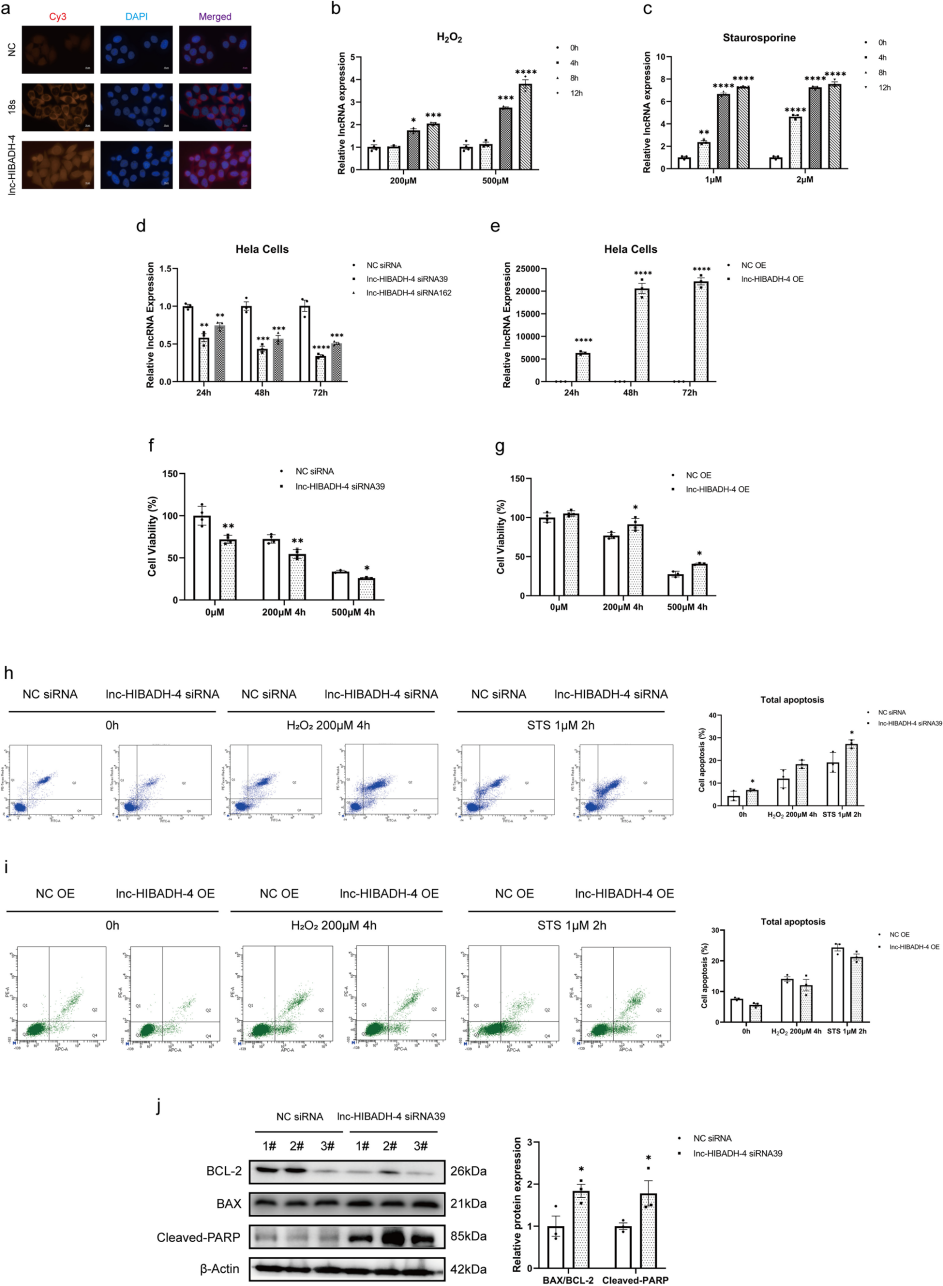
Lnc-HIBADH-4 knockdown inhibited cell proliferation and promotes apoptosis. **a** Cellular localization of lnc-HIBADH-4 in HeLa cells by RNA FISH (scale bar: 10 μm). **b** The expression of lnc-HIBADH-4 RNA in HeLa cells stimulated by H_2_O_2_ (H_2_O_2_ 200 μM 4 h versus Control, *P < 0.05; H_2_O_2_ 200 μM 12 h versus Control, ***P < 0.001; H_2_O_2_ 500 μM 4 h versus Control, ***P < 0.001; H_2_O_2_ 500 μM 12 h versus Control, ****P < 0.0001). **c** The expression of lnc-HIBADH-4 RNA in HeLa cells stimulated by STS (STS 1 μM 2 h versus Control, **P < 0.01; STS 1 μM 4 h and 8 h versus Control, ****P < 0.0001; STS 2 μM 2 h, 4 h, and 8 h versus Control, ****P < 0.0001).** d** Transfection efficiency of lnc-HIBADH-4 siRNA (HeLa cells transfected lnc-HIBADH-4 siRNA39 and siRNA162 in 24 h, 48 h, and 72 h versus Control, **P < 0.01, ***P < 0.001, ****P < 0.0001).** e** Transfection efficiency of lnc-HIBADH-4 overexpression plasmid (HeLa cells transfected lnc-HIBADH-4 OE in 24 h, 48 h, and 72 h versus Control, ****P < 0.0001). **f**, **g** Cell viabilities in lnc-HIBADH-4 knockdown/OE by CCK-8 (groups of lnc-HIBADH-4 silencing/OE in non-stimulation, 200 μM 4 h H_2_O_2_ stimulation and 500 μM 4 h H_2_O_2_ stimulation, versus corresponding groups of control, *P < 0.05, **P < 0.01). **h**, **i** Cell apoptosis in lnc-HIBADH-4 knockdown/OE by flow cytometry (groups of lnc-HIBADH-4 silencing/OE in non- stimulation, 200 μM 4 h H_2_O_2_ stimulation, 1 μM 2 h STS stimulation, versus corresponding groups of control; *P < 0.05). **j** Western blot about the level of BAX/BCL-2, cleaved-PARP between groups of lnc-HIBADH-4 silencing and controls (*P < 0.05)

Initially, we conducted RNA FISH assays to identify subcellular localization of lnc-HIBADH-4 in HeLa and SH-SY5Y cells. Our observation revealed that lnc-HIABDH-4 was expressed both in the cytoplasm and nucleus (Fig. 3a, Supplementary Fig. 2a). Additionally, we found that the levels of lnc-HIBADH-4 were upregulated in various pathological conditions in vitro, such as increased oxidative stress induced by H_2_O_2_ or apoptosis promoted by staurosporine (STS) (Fig. 3b, c, Supplementary Fig. 2b-c). Specifically, we utilized two individual siRNAs (siRNA39 and siRNA162) to knockdown lnc-HIBADH-4 RNA expression (Fig. 3d, Supplementary Fig. 2d). In addition, we also transfected the lnc-HIBADH-4 expression vector into HeLa and SH-SY5Y cells (Fig. 3e, Supplementary Fig. 2e). Gene expression analysis revealed that HeLa cells demonstrated a more significant degree of knockdown or overexpression, so that we chose HeLa cells in further experiments to explore the mechanisms of lnc-HIBADH-4. CCK-8 assays indicated that lnc-HIBADH-4 knockdown decreased cell viability (Fig. 3f). Furthermore, we found that after stimulation by H_2_O_2_, cell survival decreased further in the lnc-HIBADH-4 siRNA group, while overexpression of lnc-HIBADH-4 enhanced cell viability (Fig. 3f, g, Supplementary Fig. 2f). In addition, we investigated the impact of lnc-HIBADH-4 on cell apoptosis under both physiological and pathological conditions. Our flow cytometry showed that knockdown of lnc-HIBADH-4 increased cell apoptosis significantly (Fig. 3h). Moreover, we also discovered that under conditions of oxidative stress (cells stimulated by H_2_O_2_) and the apoptosis-inducing effect of STS, knockdown lnc-HIBADH-4 further promoted cell apoptosis compared to the control groups (Fig. 3h). Although we did not observe that lnc-HIBADH-4 overexpression affected cell apoptosis in HeLa cells, gain of lnc-HIBADH-4 partially protected cells from apoptosis under the stimulation of H_2_O_2_ and STS (Fig. 3i). Meanwhile, silencing of lnc-HIBADH-4 decreased the expression level of BCL-2 but increased the level of BAX, and cleaved-PARP (Fig. 3j, Supplementary Fig. 2g), while overexpression of lnc-HIBADH-4 decreased the level of cleaved-PARP (Supplementary Fig. 2h). Therefore, our results indicated that knockdown of lnc-HIBADH-4 promoted cell apoptosis and inhibited proliferation, highlighting the crucial role of lnc-HIBADH-4 in maintaining a healthy physiological environment and preventing the adverse effects of pathological factors.

### Knockdown lnc-HIBADH-4 Inhibits Lysosomal Function by Downregulated CTSD

**Fig. 4:**
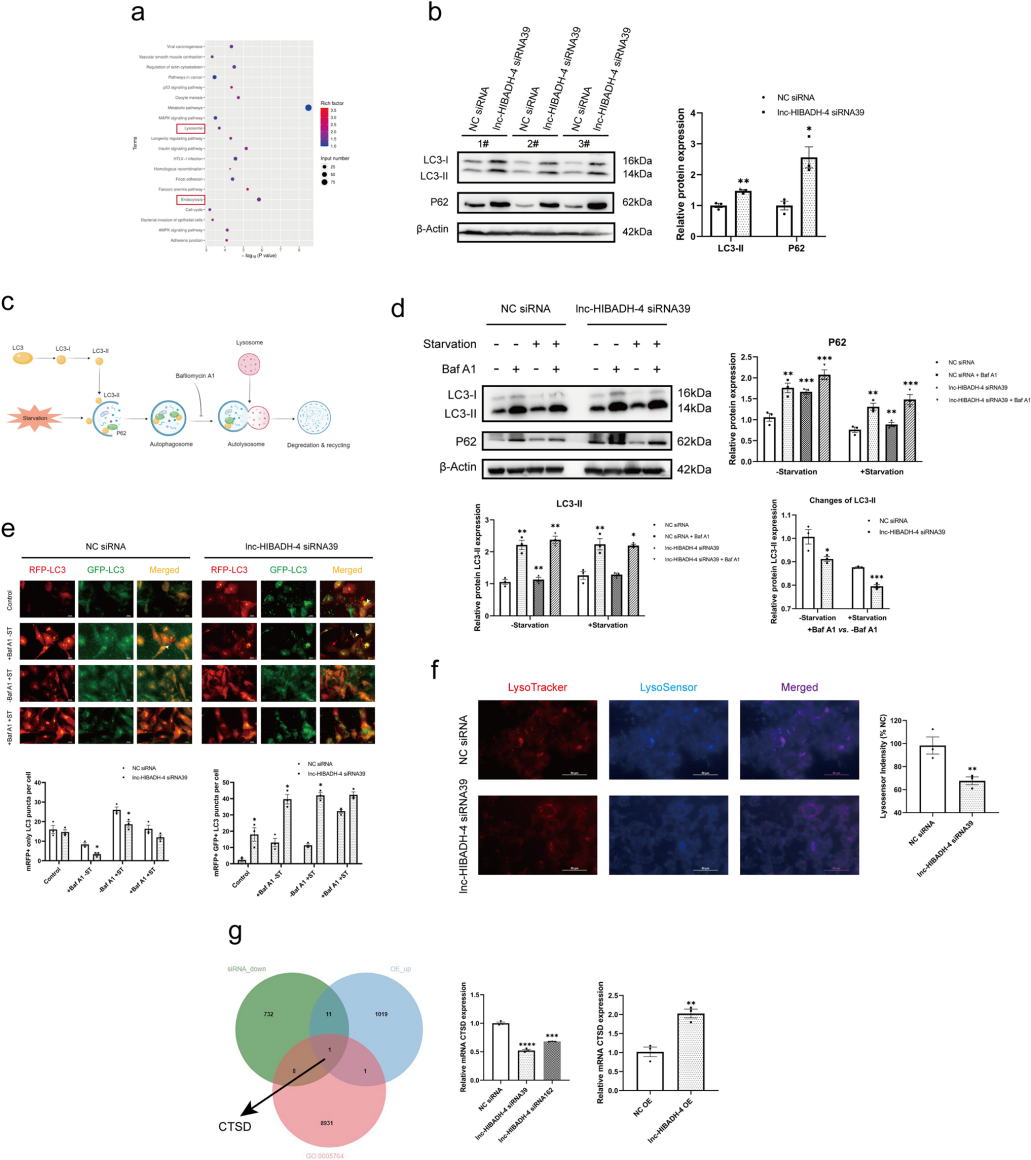
Knockdown lnc-HIBADH-4 inhibits lysosomal function by down-regulated CTSD. **a** KEGG analysis about HeLa cell transcriptome sequencing of lnc-HIBADH-4 silencing. **b** Western blot about the levels of LC3-II and P62 between groups of lnc-HIBADH-4 knockdown and Controls (*P < 0.05, **P < 0.01). **c** Diagram about the function of Baf A1 and EBSS in the autophagy-lysosome pathway. **d** Western blot about the levels of LC3-II and P62 between groups of lnc-HIBADH-4 knockdown and Controls in the condition of Baf A1 and EBSS (NC siRNA + Baf A1, lnc-HIBADH-4 siRNA, lnc-HIBADH-4 siRNA + Baf A1 versus NC siRNA separately in the condition of ± starvation; changes of LC3-II: the ratio of LC3-II in baf A1 groups to non-baf A1 groups; Baf A1: 200 nM 4 h, EBSS: 4 h). **e** mRFP-GFP-LC3 fluorescent probe diagrams of HeLa cells by lnc-HIBADH-4 knockdown (long red arrow: mRFP + only spots; short white arrow: mRFP + GFP + spot; Baf A1: 200 nM 4 h, EBSS: 4 h; *P < 0.05; scale bar: 20 μm). **f** LysoTracker and LysoSensor probe diagrams of HeLa cells by lnc-HIBADH-4 knockdown (LysoSensor indensity: the ratio of LysoSensor fluorescence intensity to LysoTracker fluorescence intensity; scale ratio: 50 μm). **g** The intersection between RNA-seq containing downregulated genes of lnc-HIBADH-4 knockdown, upregulated genes of lnc-HIBADH-4 OE and lysosomal genes. RT-qPCR results of CTSD mRNA expression by lnc-HIBADH-4 knockdown and OE (**P < 0.01, ****P < 0.0001)

To explore the biological function of lnc-HIBADH-4, we performed transcriptome sequencing of HeLa cells transfected by lnc-HIBADH-4 siRNA39. Our analysis revealed that the knockdown of lnc-HIBADH-4 led to the enrichment of multiple pathways, including p53 signaling pathway, lysosome, longevity regulating pathway (Fig. 4a). Given that KEGG analysis focused on both lysosome and endocytosis, and the autophagy-lysosomal pathway is critical in pathophysiology of ALS [22], we first explored the role of lnc-HIBADH-4 in autophagy. We discovered that lnc-HIBADH-4 knockdown significantly increased the level of LC3-II, and interestingly, the level of P62 was also increased (Fig. 4b, Supplementary Fig. 3a). Meanwhile, overexpression of lnc-HIBADH-4 displayed downregulated levels of LC3-II and P62 (Supplementary Fig. 3b). Since autophagy is a dynamic process in physiology, we applied bafilomycin A1 (baf A1) and Earle’s balanced salt solution (EBSS) to regulate the process of autophagy (Fig. 4c). Baf A1 mainly inhibits autophagy in late stage by blocking the fusion of the autophagosome with the lysosome and inhibiting acidification and protein degradation in the lysosomes. EBSS, functioned as starvation, activates the process of autophagy-lysosome pathway [32]. We discovered that silencing lnc-HIBADH-4 significantly increased the level of LC3-II in the absence of baf A1. Even though the stimulation of baf A1 increased the level of LC3-II both in control and silenced groups, the change of LC3-II ($\frac{LC3-II\;level\;in\;the\;stimulation\;of\;baf\;A1}{LC3-II\;level\;in\;the\;absence\;of\;baf\;A1}$) was lower in the silenced group than the control group (Fig. 4d, Supplementary Fig. 3c). Meanwhile, the groups of lnc-HIBADH-4 OE exhibited the opposite (Supplementary Fig. 3d). This suggests that the silencing of lnc-HIBADH-4 may suppress the autophagic flux. Moreover, we provided an mRFP-GFP-LC3 vector transfected U2OS cell model to observe the process of autophagosome and autolysosome. In the silenced group, mRFP + GFP + double-positive spots increased compared to the control groups, while the spots of mRFP + only significantly decreased (Fig. 4e). Therefore, we hypothesized that lysosomal disfunctions occur in knockdown lnc-HIBADH-4, even in the initiation and promotion of the autophagy process. Subsequently, we found that the groups with lnc-HIBADH-4 silencing showed fewer LysoSensor + cells in LysoTracker + cells compared to the control groups (Fig. 4f), whereas the overexpression groups showed the opposite (Supplementary Fig. 3e). The combination of LysoTracker and LysoSensor probes verified that knockdown of lnc-HIBADH-4 led to lysosomal disfunctions. Taken together, we concluded that lnc-HIBADH-4 participates in maintaining the dynamic balance of lysosomal functions.

Considering the association of knockdown lnc-HIBADH-4 with lysosomal disfunctions, we further explored whether lnc-HIBADH-4 affects lysosomal-associated genes based on transcriptome sequencing results of HeLa cells. We identified the common gene CTSD from the intersection of lnc-HIBADH-4 siRNA, OE, and lysosomal gene databases (https://www.gsea-msigdb.org/gsea/msigdb/cards/LYSOSOME) (Fig. 4g, Supplementary Fig. 3f). Subsequently, we conducted RT-qPCR to validate the changes of lysosomal genes in lnc-HIBADH-4 siRNA and OE groups and specified that CTSD changed simultaneously with lnc-HIBADH-4 (Fig. 4g). We also supplemented the expression of several lysosomal-associated genes, such as CTSB, LAMP1, and LAMP2, which were unchanged between lnc-HIBADH-4 siRNA/OE and their control groups (Supplementary Fig. 3g-h). Based on our findings, we deduced that knockdown of lnc-HIBADH-4 downregulates CTSD and affects the normal function of lysosomes.

### Lnc-HIBADH-4 Sponges miR-326 to Upregulate CTSD Expression and Regulate the Cell Proliferation and Apoptosis

**Fig. 5:**
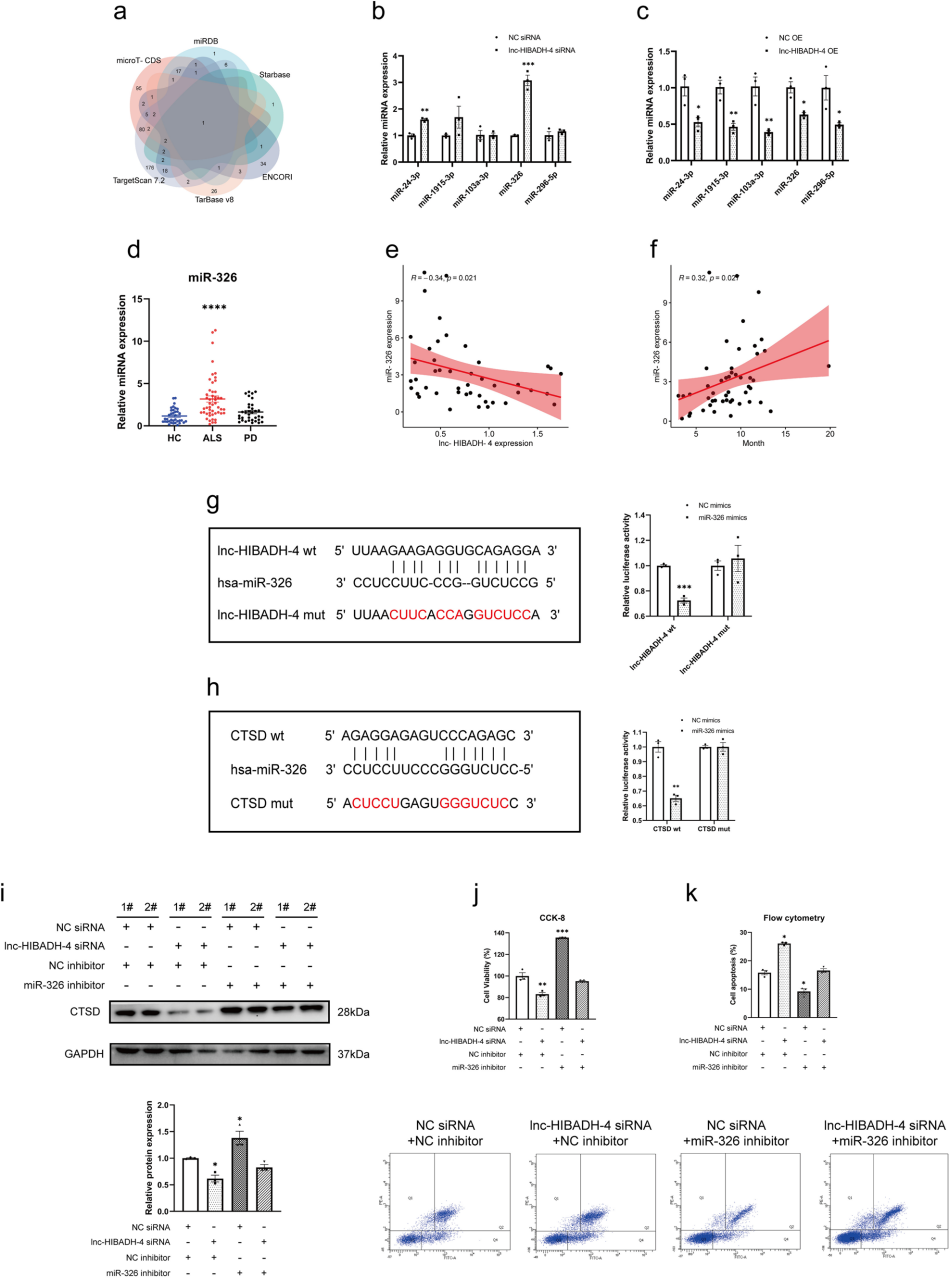
Lnc-HIBADH-4 effects cell proliferation and apoptosis by sponging miR-326 to upregulate CTSD expression. **a** Intersection of miRNAs from prediction websites of CTSD-target miRNAs. **b**, **c** The expression of selected miRNAs by lnc-HIBADH-4 knockdown and OE (*P < 0.05, **P < 0.01, ***P < 0.001). **d** miR-326 expression in sALS patients of validation cohort (****P < 0.0001). **e** Pearson correlation of the expression of lnc-HIBADH-4 and miR-326 in sALS patients. **f** Pearson correlation of the expression of miR-326 and disease duration (month). **g**, **h** Diagrams of binding sites between lnc-HIBADH-4/CTSD and miR-326, and dual luciferase reporter assays on the lnc-HIBADH-4/CTSD-wt and miR-326 mimics (**P < 0.01, ***P < 0.001). **i** Rescue western blot of CTSD protein level when transfected lnc-HIBADH-4 siRNA and miR-326 inhibitor simultaneously (*P < 0.05). **j**, **k** CCK-8 of cell proliferation and flow cytometry of cell apoptosis when transfected lnc-HIBADH-4 siRNA and miR-326 inhibitor simultaneously (*P < 0.05, **P < 0.01, ***P < 0.001)

To explore the function of lnc-HIBADH-4 in lysosome and cell homeostasis, we speculated that whether lnc-HIBADH-4 exerts as a microRNA sponge to regulate target genes, since ceRNA mechanisms were the ubiquitous function of lncRNA and participate in the pathophysiology of ALS [16, 17, 33]. Therefore, it is possible that lnc-HIBADH-4 regulates CTSD through miRNAs sponging. We intersected the predicted miRNAs of CTSD from five bioinformatics websites (ENCORI, microT-CDS, TargetScan 7.2, TarBase v8, miRDB, and Starbase) (Fig. 5a). We selected 10 miRNAs that were common in at least four databases and predicted the possible binding position of lnc-HIBADH-4 using RNA22 v2 (Supplementary Table 5). We then chose 5 miRNAs (miR-24-3p, miR-1915-3p, miR-103a-3p, miR-326, miR-296-5p) with the top maximum binding energy for further analysis. RT-qPCR showed that miR-24-3p and miR-326 were upregulated in knockdown lnc-HIBADH-4 and downregulated in overexpressed lnc-HIBADH-4 (Fig. 5b, c). We detected the expressions of these two miRNAs in ALS patients and found that miR-326 significantly increased in sALS patients, while miR-24-3p showed no significance between sALS patients and HC (Fig. 5d, Supplementary Fig. 4a). Pearson correlation analysis showed that there was moderately negative correlation between lnc-HIBADH-4 and miR-326 expression in ALS patients (Fig. 5e). We also found that the expression of miR-326 was positively correlated with disease duration (Fig. 5f). However, there was no association between miR-326 and other clinical characteristics, such as disease severity and survival probability (Supplementary Fig. 4b-f). Furthermore, luciferase assays showed that lnc-HIBADH-4-wt cells exhibited lower luciferase activity when cells were transfected by miR-326 mimics. Lnc-HIBADH-4-mut-transfected cells did not respond to miR-326 (Fig. 5g).

We then investigated the association between miR-326 and CTSD. We discovered that CTSD-wt-transfected cells were also remained lower luciferase activity with miR-326, while the results between CTSD-mut group and controls showed no significant difference (Fig. 5h). Moreover, miR-326 regulated the expression and level of CTSD mRNA and protein (Supplementary Fig. 4g-j). We also demonstrated that the protein level of CTSD was decreased by lnc-HIBADH-4 knockdown, while miR-326 inhibitor rescued the level of CTSD (Fig. 5i). Besides, CCK-8 and flow cytometry analysis showed that miR-326 inhibitor also rescued the apoptosis and inhibition of proliferation induced by lnc-HIBADH-4 silencing (Fig. 5j, k). Meanwhile, miR-326 mimics showed the opposite (Supplementary Fig. 4k-l). As shown above, we speculated that lnc-HIBADH-4 maintains cell viability and apoptosis by the mechanisms of ceRNA, such as sponging miR-326 to upregulate CTSD expression.

## Discussion

**Fig. 6:**
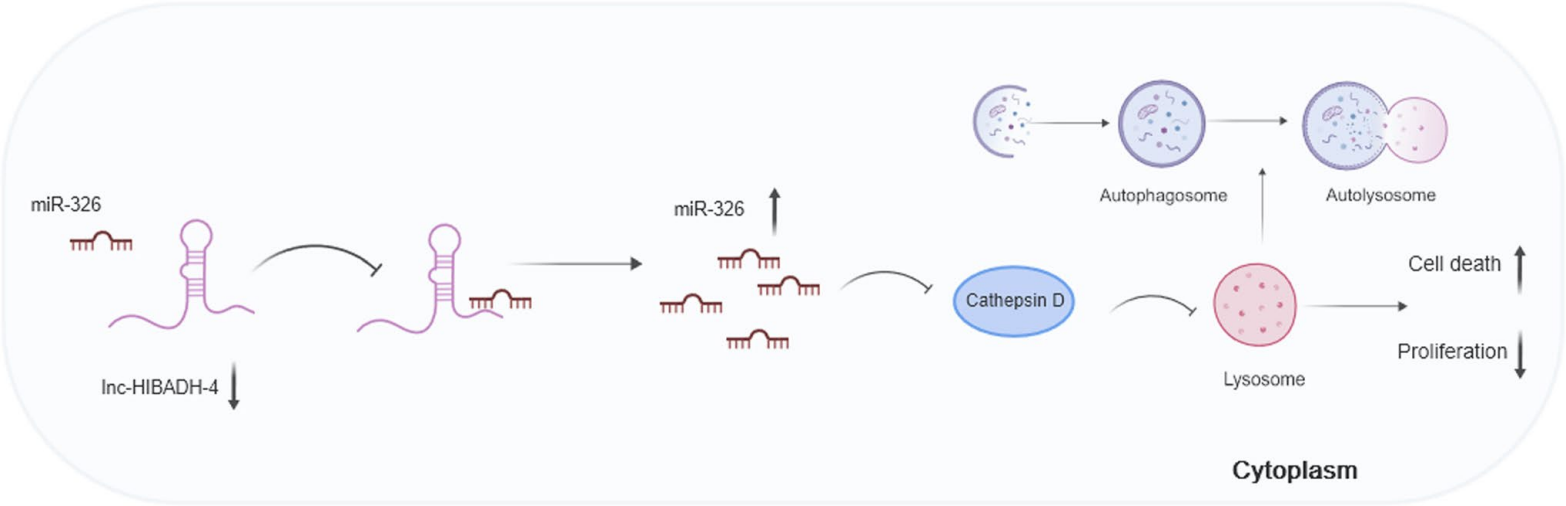
Graphic abstract about the function of lnc-HIBADH-4 in cells

ALS is a complex disease believed to be caused by environmental and genetic factors, but the specific mechanism remains elusive [34]. Non-coding RNA, a large class of RNA, plays an important role in tumorigenesis, aging, and may serve as a potential drug target [35, 36]. Previous study of microarray assay for peripheral blood mononuclear cells of sALS and HC showed that the expression of RNAs that changed between sALS and HC were mostly non-coding RNA, especially lncRNAs [37]. These results suggest that non-coding RNA may play an important role in the pathogenesis of ALS. However, few studies have explored the function of lncRNAs in ALS experimentally [38, 39]. Our study revealed that lnc-HIBADH-4 is significantly downregulated in ALS patients and correlates with disease severity and overall survival, which further confirm the role of lncRNAs in ALS. Lnc-HIBADH-4 regulates lysosomal function by competitively sponging miR-326, which increases CTSD expression and participates in proliferation and apoptosis (Fig. 6).

We identified and validated lnc-HIBADH-4 as one of the most significantly downregulated lncRNAs in ALS patients. Several miRNAs [15, 19], including miR-181, have been found to be differentially expression in peripheral blood cells and may serve as novel biomarkers when combined with NfL [14]. Although several studies have investigated the mechanisms of lncRNAs in the pathogenesis of ALS, their potential as diagnostic and prognostic biomarkers remain unknown [39–41]. Our study demonstrated that decreased lnc-HIBADH-4 expression was negatively correlated with disease severity and survival in ALS patients. Lnc-HIBADH-4 is an intronic lncRNA of JAZF1, located in chromosome 7, and its biological function in ALS has not been explored previously. Therefore, it is essential to investigate the role of lnc-HIBADH-4 in ALS pathogenesis.

We found that silencing lnc-HIBADH-4 resulted in increased cell apoptosis and decreased cell proliferation. Although the pathogenesis of ALS is considered as non-diving neuron death, we speculated that lnc-HIBADH-4 on cell proliferation was a general effect. Furthermore, we focused on the level of cell proliferation under conditions of oxidative stress and apoptosis-inducing stimuli, indicating the protective role of lnc-HIBADH-4 in cells and detrimental in lnc-HIBADH-4 downregulation. These findings suggest that decreased expression of lnc-HIBADH-4 is detrimental to cells and may contribute to the pathogenesis of ALS. We further investigated the mechanisms underlying the effects of lnc-HIBADH-4 on cell function and observed that downregulation of lnc-HIBADH-4 inhibited the autophagy-lysosomal pathway, particularly by impairing the formation of autophagolysosomal and inducing lysosomal dysfunction. Lysosomes play a crucial role in the autophagy pathway by degrading cytosolic proteins and organelles [4]. CTSD belongs to cathepsin family, a group of proteases involved in lysosomal function [27]. In addition to exerting intralysosomal proteolysis in cells [42], cathepsins take part in in homeostasis of lysosomal pH by regulating calcium, hydrion channels [43, 44]. CTSD is widely expressed in neurons and is essential for the clearance of protein aggregates and neuroprotection [27, 45]. Decreased expression of CTSD in neurons results in the accumulation of proteins and impairs lysosomal activity, particularly in neurodegenerative diseases such as ALS [27, 46, 47]. Studies have shown that CTSD levels are reduced in spinal motor neurons of TDP-43 knockout mice following lysosomal dysfunction, while in sALS and *SOD1*^*G93A*^ mice, CTSD mRNA expression increases gradually with disease progression [48, 49]. Although the spinal cord of *SOD1*^*G93A*^ mice showed increased expressions of CTSD, but CTSD were almost cleaved and without normal function in lysosome [50]. These findings demonstrate the critical role of CTSD in lysosomal function in ALS and suggest that the expression of CTSD mRNA may increase in response to lysosomal dysfunction in ALS patients and models. Both decreased expression and dysfunction of CTSD can impact the pathophysiological process of ALS, underscoring the importance of maintaining normal levels and function of CTSD in neurons. Therefore, developing therapies that target CTSD and other components of the lysosomal pathway holds promise for treating ALS and other neurodegenerative diseases.

Our research found that knockdown lnc-HIBADH-4 resulted in decreased expression of CTSD and lysosomal dysfunction. However, the mechanism underlying the link between lnc-HIBADH-4 and CTSD expression required further investigation. Considered that lnc-HIBADH-4 is located in the cytoplasm primarily, where miRNAs frequently interact with lncRNAs and mRNAs, therefore, we examined the potential role of miRNAs in mediating the relationship between lnc-HIBADH-4 and CTSD. We identified miR-326 as a potential bridge between lnc-HIBADH-4 and CTSD. We discovered a negative correlation between miR-326 and lnc-HIBADH-4 expression levels, and found that miR-326 was significantly upregulated in ALS patients, consistent with prior research conducted an Italian group [51]. MiR-326 has been suggested as a potential biomarker for neurodegenerative diseases and has been shown to inhibit cell growth and promote cell death in various disease models [52–55]. Studies have reported different roles for miR-326 in autophagy, with different target genes being identified for miR-326 [56, 57]. To our knowledge, this is the first study to identify a relationship between miR-326 and lysosomal function. Taken together, our findings suggest that lnc-HIBADH-4/miR-326 represents a potential therapeutic target for regulating CTSD expression in ALS. Future studies aimed at further elucidating the roles of lnc-HIBADH-4 and miR-326 in the pathogenesis of ALS may lead to the development of novel therapeutic approaches for the treatment of this devastating disease.

Our study has several limitations that should be addressed in the future research. Firstly, while we focused on the expression of lnc-HIBADH-4 in ALS patients, there are numerous other lncRNAs that are differentially expressed in ALS. It remains unclear whether other lncRNAs are involved in the pathogenesis of the disease [11]. Secondly, our investigation of lnc-HIBADH-4 was conducted in vitro, using HeLa cells as a model system. However, due to the low conservation of lnc-HIBADH-4 (no expression in mouse and other non-primates), it is necessary to study its role in vivo (primates) and in motor neuron cells derived from induced pluripotent stem cells (iPSCs) of ALS patients. Moreover, future studies could investigate the upstream regulatory factors of lnc-HIBADH-4 to identify potential therapeutic targets for maintaining normal levels of lnc-HIBADH-4 expression in cells, which may help prevent the occurrence and progression of ALS.

## Conclusion

In summary, our study demonstrated that lnc-HIBADH-4 was downregulated in ALS patients and was associated with disease severity and overall survival. Loss of lnc-HIBADH-4 led to cell apoptosis and inhibited cell proliferation. Specifically, downregulation of lnc-HIBADH-4 reduced its ability to sponge miR-326, resulting in dysregulation of the miR-326/CTSD pathway and further contributing to lysosomal dysfunction. Our findings suggest that lnc-HIBADH-4 may serve as a potential biomarker for ALS, and maintaining its expression and CTSD activity may represent a promising pharmacological protective intervention against neurodegeneration in ALS. Further studies are needed to determine the role of the lnc-HIBADH-4/miR-326/CTSD pathway in vivo so that pharmacological agents can be developed to restore this vital process and protect neurons from degeneration in ALS. Such investigations could lead to the development of novel therapies for treating ALS and other neurodegenerative diseases.

### Supplementary Information

Below is the link to the electronic supplementary material.

Supplementary file1 (DOCX 4.88 MB)

## Data Availability

All data in this study are in this study and supplementary materials.

## Abbreviations

ALS: Amyotrophic lateral sclerosis
lncRNA: Long non-coding RNA
PD: Parkinson’s disease
HC: Healthy control
ALSFRS-R: ALS Functional Rating Scale-Revised
CTSD: Cathepsin D
Baf A1: Bafilomycin A1
miRNA: MicroRNA
RT-qPCR: Reverse transcription–quantitative polymerase chain reaction
FBS: Fetal bovine serum
EBSS: Earle’s balanced salt solution
siRNA: Small interfering RNA
OE: Overexpression
GAPDH: Glyceraldehyde-3-phosphate dehydrogenase
BCL-2: B-cell lymphoma-2
BAX: BCL-2-associated X
LC3: Microtuble-associated protein light chain 3
SQSTM1/P62: Sequestosome 1
