# Supplementary material for: Lnc-HIBADH-4 Regulates Autophagy-Lysosome Pathway in Amyotrophic Lateral Sclerosis by Targeting Cathepsin D
Source: Mol Neurobiol. 2023 Dec 22;61(7):4768–82. doi: 10.1007/s12035-023-03835-5 (PMC11236912; doi:10.1007/s12035-023-03835-5)
Supplement: Supplementary file 1 — Supplementary file1 (DOCX 4.88 MB) [file 12035_2023_3835_MOESM1_ESM.docx]

**Supplementary materials**

**Table legends**

**Supplementary Table 1** Detailed demographic and clinical characteristics of cohorts

**Supplementary Table 2** Primers of lncRNAs and mRNA in RT-qPCR

**Supplementary Table 3** Information of primary antibodies

**Supplementary Table 4** Information of selected lncRNAs in Microarray

**Supplementary Table 5** Top 10 miRNAs targets CTSD from prediction websites and their potential targets to lnc-HIBADH-4

**Figure legends**

**Supplementary Figure 1** **The correlation between lnc-PROM1-2 and clinical characteristics of sALS.**

**a.** Lnc-PROM1-2 expression in sALS patients with different age of onset (mean ± SEM, **P* < 0.05 in sALS with age of onset > 50y versus HC > 50y). **b.** Lnc-PROM1-2 expression in sALS patients with different age of diagnosis (mean ± SEM, **P* < 0.05 in sALS with age of diagnosis > 50y versus HC > 50y). **c.** Lnc-PROM1-2 expression in sALS patients with disease duration (mean ± SEM, **P* < 0.05 in patients with more than 10 months course of ALS versus HC). **d.** Lnc-PROM1-2 expression in sALS patients with different site of onset (mean ± SEM). **e.** Correlation analysis of lnc-PROM1-2 expression and ALSFRS-R score (Pearson correlation analysis). **f.** Survival analysis of sALS patients with relatively low and high expression of lnc-PROM1-2 (Kaplan-Meier estimation and the log-rank test, the expression of lnc-PROM1-2 lower than mean lnc-PROM1-2 expression was defined as relatively low expression group, and vice versa).

**Supplementary Figure 2 Lnc-HIBADH-4 knockdown inhibited cell proliferation and promotes apoptosis.**

**a.** Cellular localization of lnc-HIBADH-4 in SH-SY5Y cells by RNA FISH. **b.** The expression of lnc-HIBADH-4 RNA in SH-SY5Y cells stimulated by H_2_O_2_ (H_2_O_2_ 200μM 4h versus Control, **P* < 0.05; H_2_O_2_ 200μM 12h versus Control, ****P* < 0.001; H_2_O_2_ 500μM 2h versus Control, ***P* < 0.01; H_2_O_2_ 500μM 4h versus Control, ***P* < 0.01; H_2_O_2_ 500μM 12h versus Control, *****P* < 0.0001). **c.** The expression of lnc-HIBADH-4 RNA in SH-SY5Y cells stimulated by STS (STS 1μM 2h versus Control, ****P* < 0.001; STS 1μM 4h versus Control, **P* < 0.05; STS 2μM 2h versus Control, ****P* < 0.001; STS 2μM 4h versus Control, *****P* < 0.0001). **d.** Transfection efficiency of lnc-HIBADH-4 siRNA (SH-SY5Y cells transfected lnc-HIBADH-4 siRNA39 and siRNA162 in 24h, 48h and 72h versus Control, **P* < 0.05, ***P* < 0.01). **e.** Transfection efficiency of lnc-HIBADH-4 overexpression plasmid (SH-SY5Y cells transfected lnc-HIBADH-4 OE in 24h, 48h and 72h versus Control, *****P* < 0.0001). **f.** Cell viability of lnc-HIBADH-4 knockdown (siRNA162) by CCK-8 (**P* < 0.05, ***P* < 0.01). **g.** Western blot about the level of cleaved-PARP between lnc-HIBADH-4 knockdown and controls (**P* < 0.05). **h**. Western blot about the level of BAX/BCL-2, cleaved-PARP between groups of lnc-HIBADH-4 OE and controls (**P* < 0.05).

**Supplementary Figure 3 Knockdown lnc-HIBADH-4 inhibits lysosomal function by down-regulated CTSD.**

**a-b.** Western blot about the levels of LC3-II and P62 between groups of lnc-HIBADH-4 knockdown (siRNA162)/OE and Controls (**P* < 0.05, ***P* < 0.01). **c-d.** Western blot about the levels of LC3-II and P62 between groups of lnc-HIBADH-4 knockdown (siRNA162)/OE and Controls in the condition of Baf A1 and EBSS (Changes of LC3-II: the ratio of LC3-II in baf A1 groups to non-baf A1 groups; Baf A1: 200nM 4h, EBSS: 4h; **P* < 0.05, ***P* < 0.01). **e.** LysoTracker & LysoSensor probe diagrams of HeLa cells by lnc-HIBADH-4 OE (LysoSensor indensity: the ratio of LysoSensor fluorescence intensity to LysoTracker fluorescence intensity; scale ratio: 50μm). **f.** The intersection between RNA-seq containing upregulated genes of lnc-HIBADH-4 knockdown, downregulated genes of lnc-HIBADH-4 OE and lysosomal genes. **g-h.** RT-qPCR results of CTSB, LAMP1, LAMP2 mRNA expression by lnc-HIBADH-4 knockdown and OE (ns: no significance).

**Supplementary Figure 4 Lnc-HIBADH-4 effects cell proliferation and apoptosis by sponging miR-326 to upregulate CTSD expression.**

**a.** miR-24-3p expression in sALS patients of validation cohort. **b.** Pearson correlation of the expression of lnc-HIBADH-4 and ALSFRS-R score in sALS patients. **c.** Survival analysis of sALS patients with relatively low and high expression of miR-326. **d-f.** Pearson correlation of the expression of lnc-HIBADH-4 and age of onset, age of diagnosis and progression rate in sALS patients. **g-h.** Western blot and RT-qPCR of CTSD protein level and expression in the condition of miR-326 inhibitor (*****P* < 0.0001). **i-j.** Western blot and RT-qPCR of CTSD protein level and expression in the condition of miR-326 inhibitor (**P* < 0.05, ***P* < 0.01). **k-l.** CCK-8 of cell proliferation and flow cytometry of cell apoptosis when transfected lnc-HIBADH-4 OE and miR-326 mimics simultaneously (***P* < 0.01, *****P* < 0.0001).

**Supplementary Table 1 Detailed demographic and clinical characteristics of cohorts**

| Characteristics | ALS | HC | PD |
| --- | --- | --- | --- |
| Number | 60 | 40 | 40 |
| Male（%） | 35（58.33%） | 20（50.00%） | 21（52.5%） |
| Age of diagnosis（year, SEM*） | 52.85（0.09） | 51.28（0.05） | 54.19（0.30） |
| Age of onset（year, SEM） | 52.16（0.09） | - | 52.79（0.29） |
| Disease duration（month, SEM） | 8.32（0.05） | - | 42.70（0.59） |
| ALSFRS-R score（SEM） | 39.75（0.09） | - | - |
| Disease progression rate（SEM）† | 1.16（0.02） | - | - |

Note: *SEM（Standard error）；† Disease progression rate = (48- ALSFRS-R score of diagnosis）/ Disease duration (month)

**Supplementary Table 2 Primers of lncRNAs and mRNA in RT-qPCR**

| Gene | Primer | Sequence |
| --- | --- | --- |
| lnc-HIBADH-4 | Forward | GGTGATGAACCACAACCCTTG |
|  | Reverse | ACTATCACTTCCCACACCCAG |
| lnc-ARL6IP1-4 | Forward | CAGGAGGCGTCTTCAGTTTG |
|  | Reverse | GGGAAGGAGCTCAACTAGGG |
| NONHSAG006133 | Forward | GCTGGTCTGCTTTGTGTGTTA |
|  | Reverse | CACTCATGACCATTGGAAAGCA |
| lnc-PROM1-2 | Forward | CCCCAAATTCTAAACTCTCAGGA |
|  | Reverse | CCTCAATTCCACCGAACTGT |
| XXbac-BPG299F13.14 | Forward | CTAGAAAAATGCCTGGCACA |
|  | Reverse | TGCCAAAACCAAACCAGTAA |
| lnc-ECH1-2 | Forward | GGCAATGTGGAGAAGGTGAAA |
|  | Reverse | TGAGCACCTACCAGACATTCA |
| GAPDH | Forward | ATGAGAAGTATGACAACAGCCTCAAGAT |
|  | Reverse | ATGAGTCCTTCCACGATACCAAAGTT |
| ACTB | Forward | TCACCATGGATGATGATATCGC |
|  | Reverse | AATCCTTCTGACCCATGCC |
| CTSD | Forward | CAGCCAGGCATCACCTTCAT |
|  | Reverse | CAGGTAGAAGGAGAAGATGT |
| CTSB | Forward | AGGTCCCAGCTTGAAGAGAAA |
|  | Reverse | CGCAGGTGTACGAAGTCCTC |
| LAMP1 | Forward | ATGGGGCTGCAGCTGAACCT |
|  | Reverse | CAGCTCCAGAGTCACCAGGT |
| LAMP2 | Forward | CGCCGATTCCTGGCTTTTG |
|  | Reverse | AAGAGCACTGATGACCACCG |

**Supplementary Table 3 Information of primary antibodies**

| Primary antibody | Molecular weight（kD） | Species | Company |
| --- | --- | --- | --- |
| β-Actin | 42 | mouse | BOSTER |
| GAPDH | 37 | rabbit | HuaBio |
| α-Tubulin | 55 | mouse | Abcam |
| Cleaved-PARP | 89 | rabbit | HuaBio |
| BCL-2 | 25 | rabbit | HuaBio |
| BAX | 21 | rabbit | HuaBio |
| LC3 | 14/16 | rabbit | Novus |
| P62 | 62 | rabbit | Abcam |
| CTSD | 28 | rabbit | HuaBio |

**Supplementary Table 4 Information of selected lncRNAs in Microarray**

| lncRNA ID | Fold change（ALS *vs*. HC） | P value | Regulation |
| --- | --- | --- | --- |
| NONHSAG006133 | 2.4273775 | 0.000746 | up |
| lnc-ARL6IP1-4 | 2.422056 | 0.020006748 | up |
| lnc-PROM1-2 | 2.392793 | 0.015917996 | down |
| lnc-HIBADH-4 | 2.358123 | 0.04595575 | down |
| XXbac-BPG299F13.14 | 2.3294568 | 0.001971965 | up |
| lnc-ECH1-2 | 2.3214803 | 0.011817011 | down |

**Supplementary Table 5 Top 10 miRNAs targets CTSD from prediction websites and their potential targets to lnc-HIBADH-4**

| Websites | miRNA | mfe (kcal/mol) binding to lnc-HIBADH-4 |
| --- | --- | --- |
| microT-CDS\|miRDB\|Starbase\|ENCORI\|TarBase v8\|TargetScan 7.2 | hsa-miR-24-3p | -24.2 |
| microT-CDS\|miRDB\|ENCORI\|TargetScan 7.2 | hsa-miR-2467-3p | -21.7 |
| microT-CDS\|miRDB\|TarBase v8\|TargetScan 7.2 | hsa-miR-1915-3p | -25.6 |
| microT-CDS\|Starbase\|ENCORI\|TarBase v8\|TargetScan 7.2 | hsa-miR-103a-3p | -23.6 |
|  | hsa-miR-107 | -22.6 |
| microT-CDS\|Starbase\|ENCORI\|TargetScan 7.2 | hsa-miR-326 | -30.7 |
|  | hsa-miR-185-5p | -19.7 |
| microT-CDS\|ENCORI\|TarBase v8\|TargetScan 7.2 | hsa-miR-296-5p | -23.6 |
|  | hsa-miR-147a | -22.7 |
| miRDB\|ENCORI\|TarBase v8\|TargetScan 7.2 | hsa-miR-574-5p | -20.9 |

**Supplementary Figure 1** The correlation between lnc-PROM1-2 and clinical characteristics of sALS.


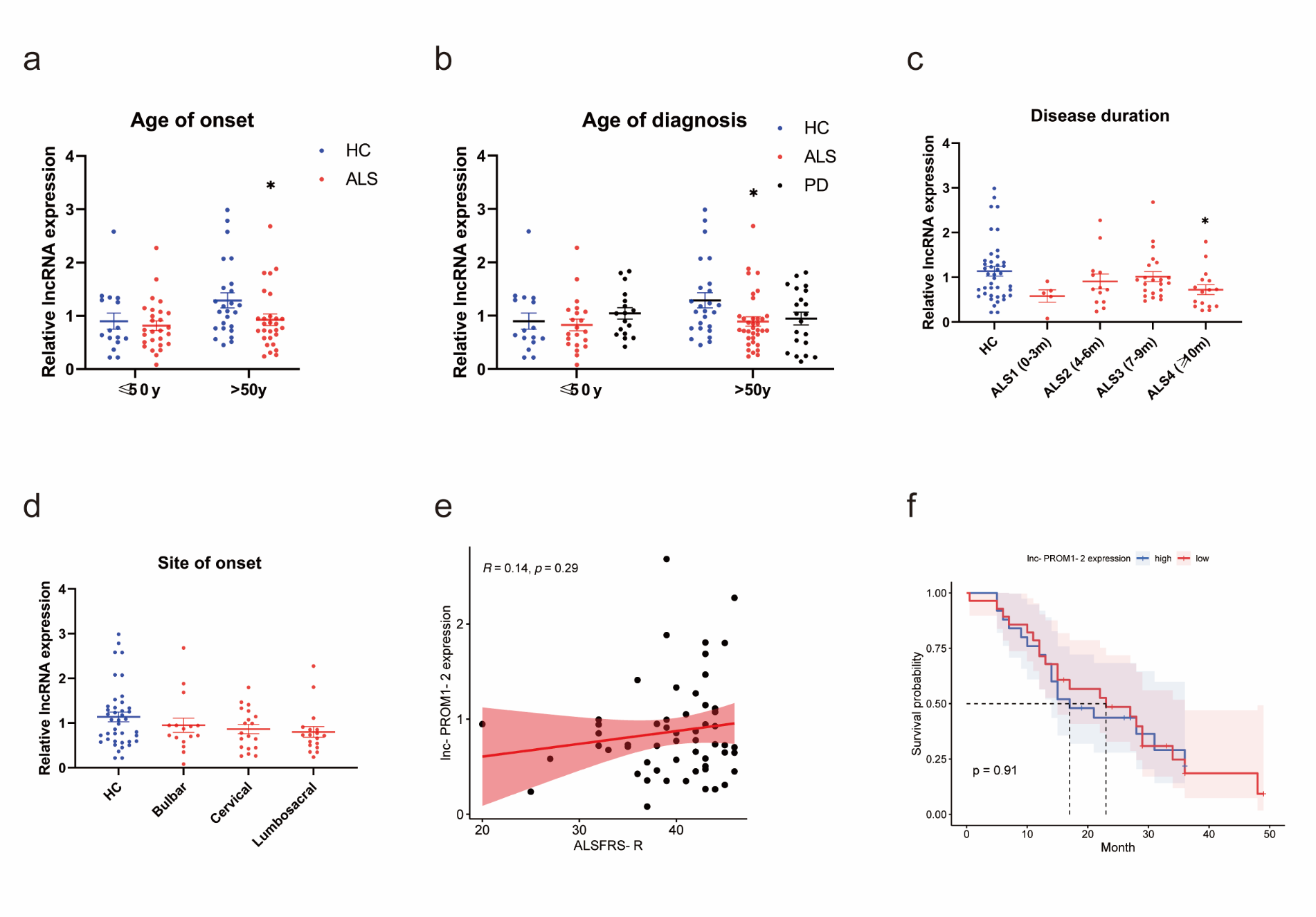


**Supplementary Figure 2** Lnc-HIBADH-4 knockdown inhibited cell proliferation and promotes apoptosis.


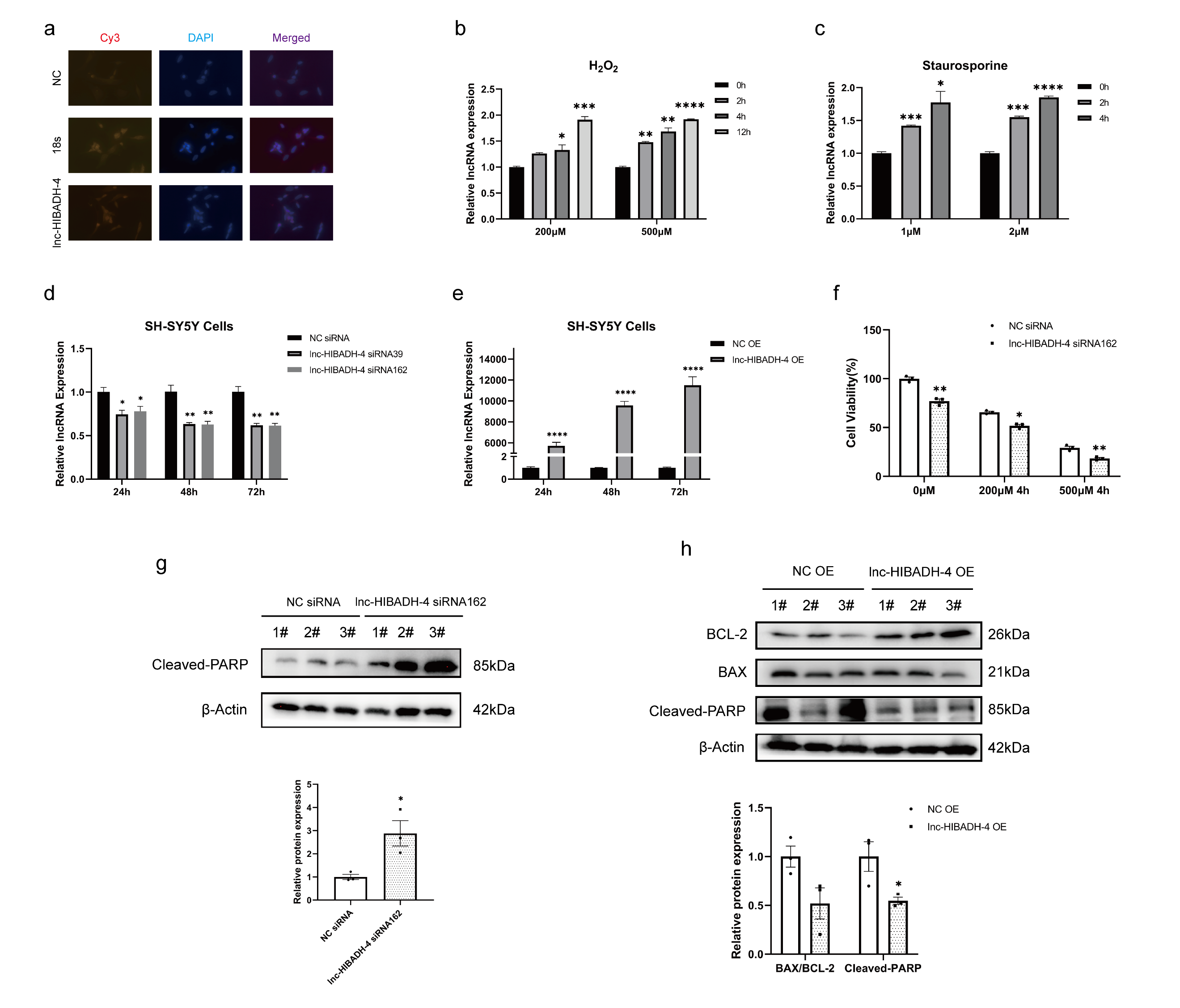


**Supplementary Figure 3** Knockdown lnc-HIBADH-4 inhibits lysosomal function by down-regulated CTSD.

**
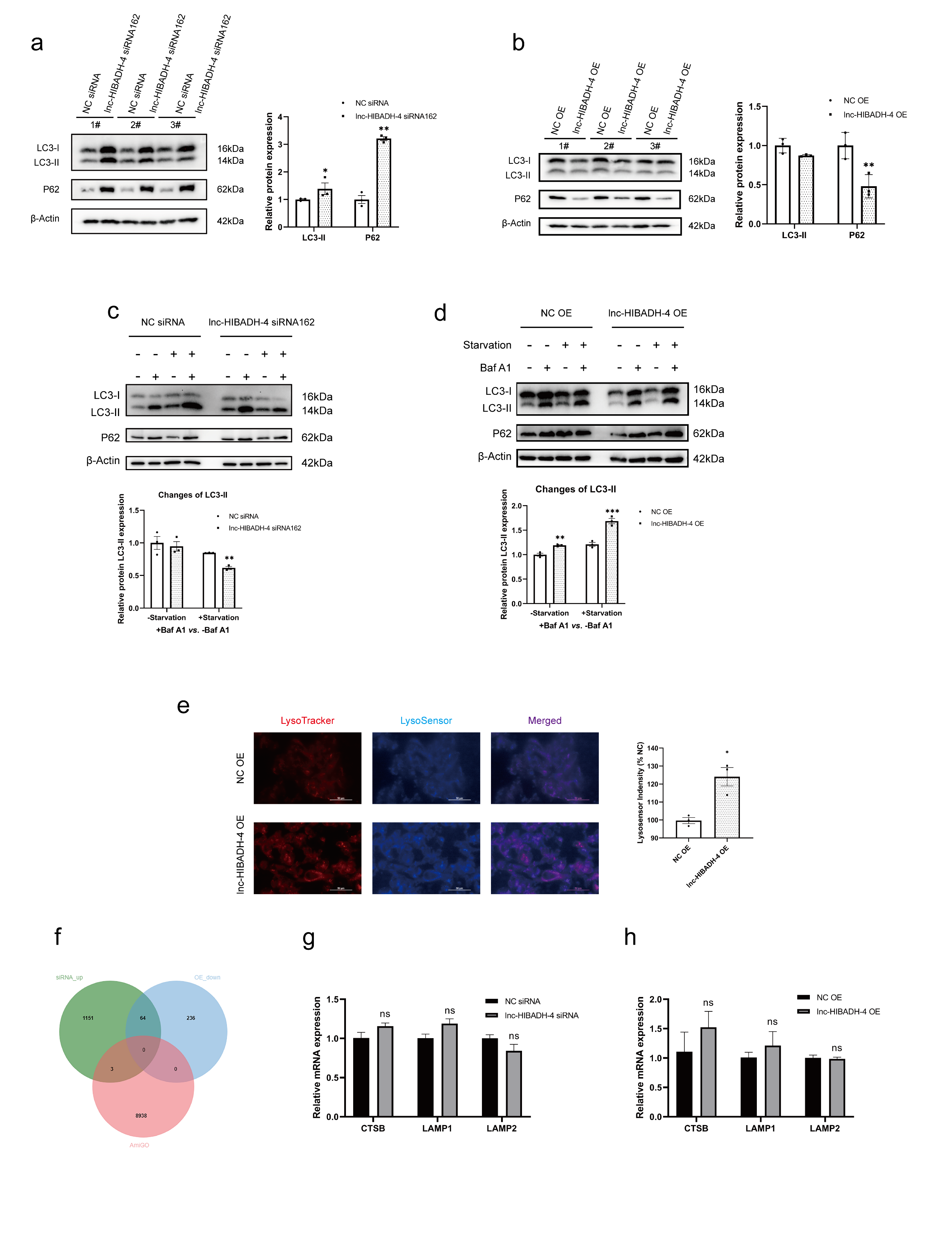
**

**Supplementary Figure 4** Lnc-HIBADH-4 effects cell proliferation and apoptosis by sponging miR-326 to upregulate CTSD expression.

**
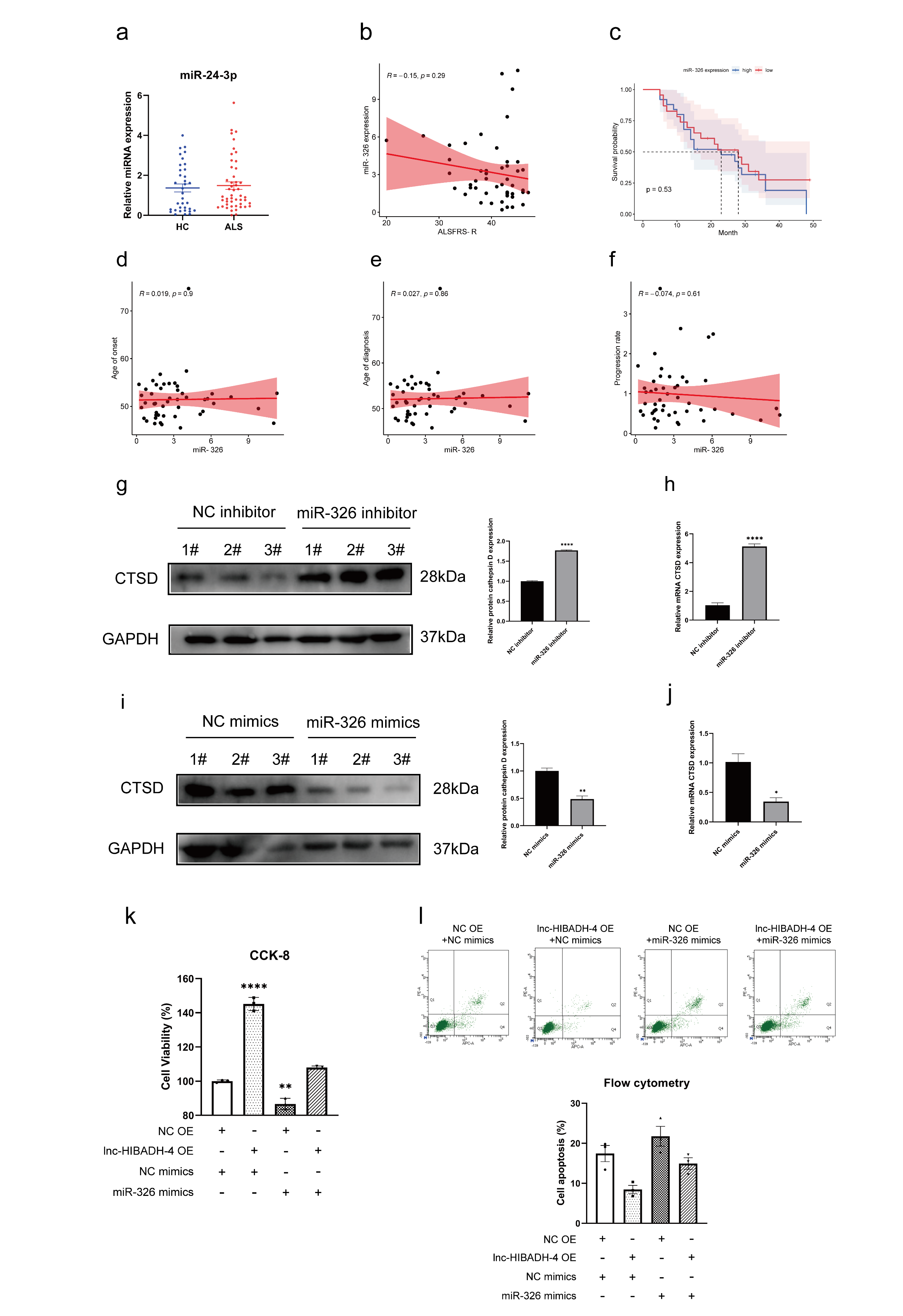
**
